# A new design approach for dual-band power amplifiers based on dual-band HCC and bandpass filter

**DOI:** 10.1038/s41598-024-51456-2

**Published:** 2024-01-15

**Authors:** Sepehr Zarghami, Mohsen Hayati

**Affiliations:** Electrical Engineering Department, Faculty of Engineering, Razi University, Kermanshah, 67149-67346 Iran

**Keywords:** Biomedical engineering, Electrical and electronic engineering

## Abstract

This paper introduces a novel design approach based on the dual-band harmonic control circuit and bandpass filter for dual-band power amplifiers. The circuit schematic of the proposed approach is constructed using four resonators and RFC inductors. The first two resonators are dedicated to controlling the second harmonics, while the third and fourth resonators serve as a harmonic blocker, allowing only the main signals to pass through to the load. Subsequently, all components are replaced by circuits based on microstrip elements, forming the proposed OMN. This OMN includes a novel wideband bias circuit, elliptically coupled resonators, and a new dual-band bandpass filter. To ensure compatibility with the transistor, a compensator line has been integrated. As a result, a dual-band power amplifier has been fabricated and measured at two operating frequencies, 2.1 GHz and 2.91 GHz. The measured values for drain efficiency, output power, and power gain at 2.1 GHz are 75.98%, 37.5 dBm, and 12.5 dB, respectively. Similarly, at 2.91 GHz, these values are 75.73%, 37.24 dBm, and 12.24 dB, respectively.

## Introduction

Dual-band microwave power amplifiers (PA) represent a critical advancement in contemporary microwave technology. These amplifiers possess the remarkable capability to amplify microwave signals in two distinct frequency bands simultaneously. In the rapidly evolving landscape of wireless communication and data transmission, they play an integral role, guided by the latest references^1–12^ and cutting-edge research. These amplifiers not only enhance signal strength but also contribute significantly to the efficiency and reliability of microwave systems.

**Figure 1:**
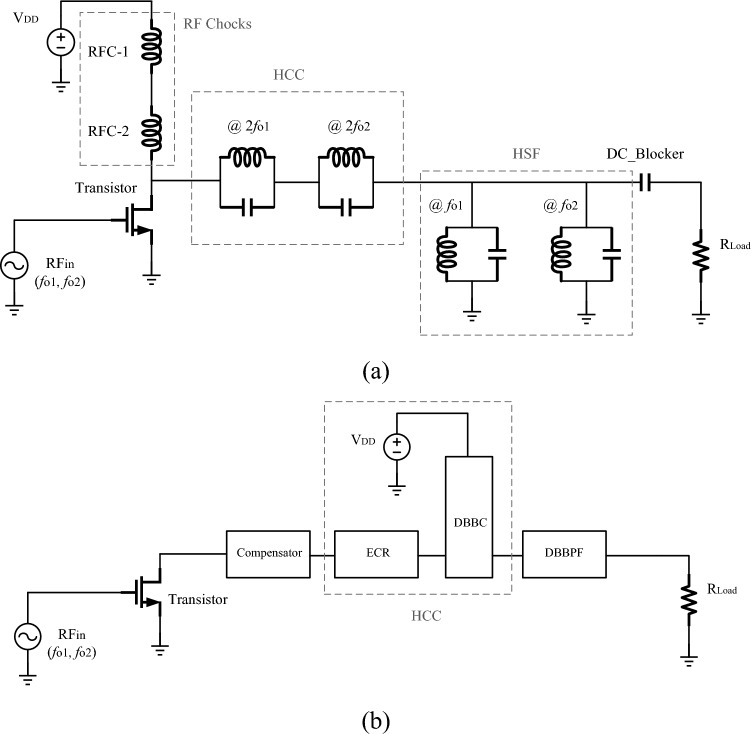
(**a**) Dual-Band PA circuit and (**b**) block diagram of the proposed dual-band PA structure.

Recently, several concepts have been employed in the design of dual-band PAs, each addressing the enhancement of a particular parameter. Designing amplifiers that can operate in two frequency bands requires the use of special harmonic control and biasing circuits. So that, unlike wideband amplifiers, they only operate in two specific bands, and other frequencies between the two bands are blocked. In this regard, the proposed schematic circuit for designing a dual-band PA based on compact elements is illustrated in Fig. 1a. In this circuit, two inductors, RFC1 and RFC2, are utilized; each resonating at one of the amplifier's operating frequencies. In^1–4^ radial stub structures are employed as biasing circuits specifically designed for dual-band amplifiers. Additionally, the proposed circuit in Fig. 1a employs two inductive-capacitive resonator circuits, each resonant at the second harmonic of amplifier’s operating frequency (@2*f*_o1_ or @2*f*_o2_), while jointly controlling both second harmonics. In essence, this section represents the harmonic control circuit (HCC), which has been previously implemented using various techniques^5–10^. The presented HCC in^5^ used two lines, each having a total length equal to a quarter-wavelength at each of the amplifier's operating frequencies. In^6^, the design incorporates 8 quarter-wavelength lines as HCC. The presented HCC in^7^ is based on the network parameter extraction method, while^8^ utilizes the precise harmonic control approach. The HCCs of^9^ and^10^ relies on impedance transformers and reactance compensation networks, and centered around a flexible network configuration with crossed transmission lines, respectively. In^13^, instead of using harmonic rejection filters, a narrow-band diplexer is used.

Furthermore, in the circuit depicted in Fig. 1a, two resonators, each comprised of an inductor and a capacitor in parallel, are evident. These resonators, resonating at the operational frequencies of the amplifier (@*f*_*o*1_ or @*f*_o2_), effectively block all harmonics, with the exception of the first harmonic. These results in the generation of pure sinusoidal signals delivered to the load. In essence, this section serves as a harmonic blocker circuit, permitting only the passage of the first harmonics. Regrettably, prior research has given less attention to this aspect of dual-band PAs, posing a significant challenge in the design of an alternative circuit for this particular function.

In previous designs, a comprehensive discussion on the replacement of specific circuits with suitable alternatives for all components of the proposed circuit in Fig. 1a has been lacking. Designing an HCC with minimal losses, utilizing fewer elements, and occupying a compact footprint poses significant challenges for designers. Additionally, a crucial aspect of the harmonic blocker circuit, beyond defining upper and lower stopbands, is ensuring isolation between the two bands and achieving a narrow bandwidth for each operating frequency of the amplifier. Consequently, designing a harmonic blocker circuit that successfully incorporates all these features simultaneously presents a significant challenge.

Therefore, this article introduces a new OMN, encompassing all components depicted in Fig. 1a, as illustrated in Fig. 1b. Initially, the HCC circuit is designed, incorporating a dual-band bias circuit and elliptically coupled resonators. Subsequently, a dual-band bandpass filter with narrow passbands is designed in place of the harmonic blocker circuit. The compensator section is integrated into the proposed OMN to align the designed circuits with the transistor. Finally, a dual-band PA is designed and fabricated using the proposed OMN, and the validity of the design process is confirmed through appropriate measured results.

## Design of the output matching network

Figure 1b illustrates the block diagram of the proposed dual-band PA, designed upon the dual-band PA circuit depicted in Fig. 1a. This innovative structure incorporates an OMN, comprising a wideband bias circuit (WB-BC), elliptically coupled resonators (ECR), a dual-band bandpass filter (DB-BPF), and a compensation line. Within this framework, WB-BC takes the place of choke inductors (RFCs), ECR replaces the second harmonic control resonators, and DB-BPF supersedes the harmonic blocker circuit. Harmonics are regulated by two sections of WB-BC and ECR, collectively forming the harmonic control circuit (HCC). Leveraging the bandpass filters' capability to eliminate the DC component from the signal, the DC blocker capacitor in the Fig. 1a circuit is omitted. The compensation section incorporates a transmission line, whose dimensions are meticulously tuned in the simulator software to achieve transistor matching with the proposed OMN. Consequently, the proposed OMN is structured into three distinct sections, and the design process for each section will be discussed individually in the subsequent sections of this paper.

## Dual-band HCC

The HCC is consisted of WB-BC and ECR to control second harmonics and prevent leakage of signals of fundamental harmonics into the DC source. The proposed circuits are designed for a dual-band PA at operating frequencies of 2.1 and 2.91 GHz.

### WB-BC

**Figure 2:**
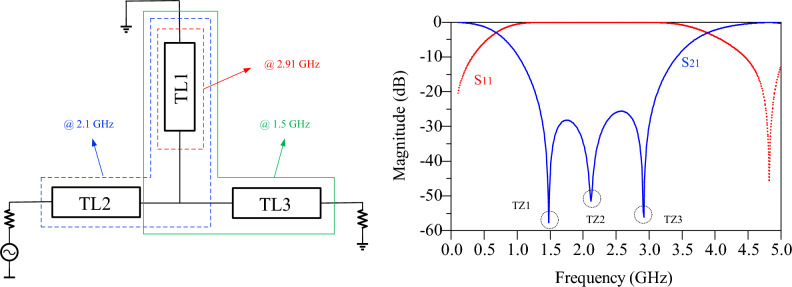
The schematic and simulation results of proposed WB-BC.

In single-band PA design, quarter-wavelength line is used instead of RFC inductor. In addition to the role of RFC (preventing high-frequency signal leakage into the DC source), this line also resonate at the even harmonic and provides short-circuit conditions for the second harmonic. As a result, a quarter-wavelength line can also be an HCC circuit, but for a single-band PAs. For the proposed dual-band PA design approach, first a WB-BC is designed, and then the control of the second harmonics will be done by the ECR circuit. The schematic diagram of the proposed WB-BC is depicted in Fig. 2. This circuit consists of one short-circuited stub and two other microstrip stubs between input and output ports that create three transmission zeros. The TL1 resonates at 2.91 GHz, the TL1 together with TL2 resonate at 2.1 GHz, and the TL1 and TL3 resonate at 1.5 GHz. As a result, a WB-BC is designed from 1.4 to 3 GHz frequency, which prevents signal leakage in the mentioned frequency range into the DC source. This bias circuit can be used in wideband and dual-band PAs, whose working band is in the operating band of the bias circuit. Figure 2 shows the simulated results of the proposed bias circuit.

### ECR

**Figure 3:**
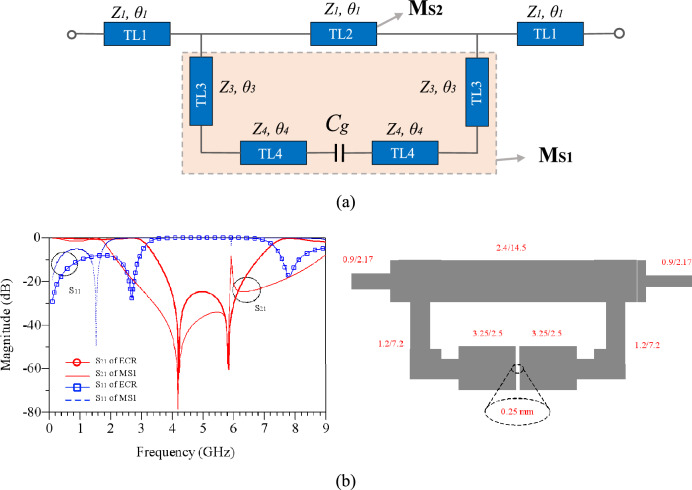
(**a**) The equivalent circuit, (**b**) layout and simulation results of ECR.

The Elliptically Coupled Resonator (ECR) serves as a novel structure designed to control the second harmonics of a dual-band PA, which has compact size and uses fewer lines. This innovative design is based on elliptical resonators that leverage a coupling effect for simultaneous control of both second harmonics. These resonators are constructed from high-impedance lines connected to open-ended low-impedance lines, strategically positioned against each other to create a coupling effect. This coupling effect doubles the number of transmission zeros within the structure. The primary purpose of this structure is to generate transmission zeros at second harmonics and fine-tune them. Figure 3 depicts the equivalent circuit and the structure of the ECR. In particular, Fig. 3a presents an approximate equivalent circuit of the ECR, where the coupling effect is modeled as a gap capacitance (C_g_). The designed ECR achieves short-circuit conditions to ground for second harmonics, effectively creating transmission zeros at frequencies of 4.2 and 5.82 GHz. In order to analyze, the *ABCD* matrix of the ECR structure has been calculated. In such a way that the *ABCD* matrix of each section is calculated separately, then by simplifying, the total *ABCD* matrix is obtained, as follows:

The *ABCD* matrix (M_TLi_) of transmission lines and the gap capacitance (M_g_) can be given as:

1 $$M_{TLi} = \left[ {\begin{array}{*{20}c} {A = \cos \theta_{i} } & {B = jZ_{i} \sin \theta_{i} } \\ {C = j{\raise0.7ex\hbox{$1$} \!\mathord{\left/ {\vphantom {1 {Z_{i} }}}\right.\kern-0pt} \!\lower0.7ex\hbox{${Z_{i} }$}}\sin \theta_{i} } & {D = \cos \theta_{i} } \\ \end{array} } \right],\;(i = 1,2,3,4,5)$$

2 $$M_{g} = \left[ {\begin{array}{*{20}c} {A = 1} & {B = \frac{1}{{j.\omega .C_{g} }}} \\ {C = 0} & {D = 1} \\ \end{array} } \right]$$

The *ABCD* matrix of the proposed main resonator can be derived as:

3 $$M_{EC} = M_{TL1} \times M_{Z2} \times M_{TL1}$$

The *ABCD* matrix M_TL1_, which is just a TL, can be obtained easily by Eq. (10) as follows:

4 $$M_{TL1} = \left[ {\begin{array}{*{20}c} {\cos \theta_{1} } & {jZ_{1} \sin \theta_{1} } \\ {j{\raise0.7ex\hbox{$1$} \!\mathord{\left/ {\vphantom {1 {Z_{1} }}}\right.\kern-0pt} \!\lower0.7ex\hbox{${Z_{1} }$}}\sin \theta_{1} } & {\cos \theta_{1} } \\ \end{array} } \right]$$

Zone 2 consists of two subsections, which *ABCD* matrix of each subsections are named as M_S1_ and M_S2_. M_S1_ consists of a one TL and its *ABCD* matrix (M_S1_) can be obtained by Eq. (13). M_S2_ can be obtained as:

5 $$M_{S1} = M_{TL3} \times M_{TL4} \times M_{g} \times M_{TL4} \times M_{TL3}$$

After obtaining M_S1_ and M_S2_, Y-matrix (Y_S1_ and Y_S2_) of M_S1_ and M_S2_ can be obtained from the *ABCD* matrix, as follows:

6 $$Y_{i} = \left[ {\begin{array}{*{20}c} {Y_{11} = {\raise0.7ex\hbox{$D$} \!\mathord{\left/ {\vphantom {D B}}\right.\kern-0pt} \!\lower0.7ex\hbox{$B$}}} & {Y_{12} = \frac{ - (AD - BC)}{B}} \\ {Y_{21} = {\raise0.7ex\hbox{${ - 1}$} \!\mathord{\left/ {\vphantom {{ - 1} B}}\right.\kern-0pt} \!\lower0.7ex\hbox{$B$}}} & {Y_{22} = {\raise0.7ex\hbox{$A$} \!\mathord{\left/ {\vphantom {A B}}\right.\kern-0pt} \!\lower0.7ex\hbox{$B$}}} \\ \end{array} } \right],\;(i = S1,S2)$$

where, *A*, *B*, *C* and *D* are the *ABCD* matrix parameters. The Y-matrix of zone 2 (Y_Z2_) can be given as:

7 $$Y_{Z2} = Y_{S1} + Y_{S2}$$

Then, the M_Z2_ can be obtained from Y_Z2_ using the following equation:

8 $$M_{Z2} = \left[ {\begin{array}{*{20}c} {A = \frac{{ - Y_{22} }}{{Y_{21} }}} & {B = \frac{ - 1}{{Y_{21} }}} \\ {C = \frac{{ - (Y_{11} Y_{22} - Y_{12} Y_{21} )}}{{Y_{21} }}} & {D = \frac{{ - Y_{11} }}{{Y_{21} }}} \\ \end{array} } \right]$$

Consequently, by Eq. (17), the *ABCD* matrix of zone 2 can be obtained.

Finally, by using (3), the *ABCD* matrix of the ECR structure is obtained. The s-parameters of ECR have been obtained by using *ABCD* matrix parameters of *M*_*EC*_ and resonating conditions^20^. Using the proposed equivalent circuit and equations of (1–8), the ECR structure is designed and simulated. The layout and simulated results are depicted in Fig. 3b. According to the results, there are two transmission zeros in the second harmonics that create a short circuit condition to ground. Figure 3b shows the simulated responses of M_S1_ zone and ECR. According to this figure, it is clear that transmission zeros are created and set by M_S1_ zone. As a result, designing the ECR circuit for any desired operating frequency, ms1 section needs to be adjusted to control second harmonics.

In fact, the ECR circuit, along with the WB-BC, is both a bias circuit and a HCC for dual-band PAs. Since the quarter-wavelength line plays the role of both RFC and second harmonic controller in the design of single-band PAs, the proposed HCC circuit creates the same functions for the dual-band PA.

## Dual-band bandpass filter

**Figure 4:**
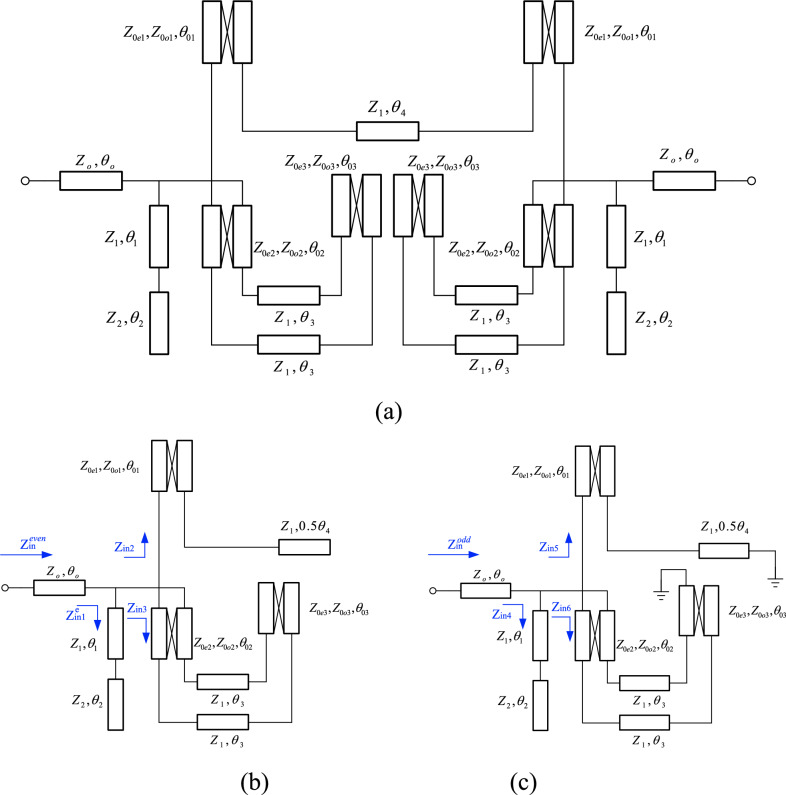
Equivalent model of the proposed DB-BPF, (**b**) even mode, (**c**) odd mode.

As previously mentioned, to selectively pass and amplify only two specific signals in the proposed dual-band PA, a dual-band bandpass filter (DB-BPF) with a new structure has been meticulously designed and simulated. The objectives of this filter align with those of the proposed amplifier, necessitating passbands at 2.1 and 2.91 GHz with a requisite level of isolation between them. Given the proximity of the two passbands, achieving a high level of suppression and an adequate isolation width is of paramount importance. The insertion and return losses at the central frequencies of the passbands are exceptionally suitable. Furthermore, the proposed filter is designed to feature lower and upper stopbands with an appropriate level of suppression. In pursuit of these goals, an equivalent model of a DB-BPF has been proposed, as illustrated in Fig. 4.

### DB-BPF analysis

**Figure 5:**
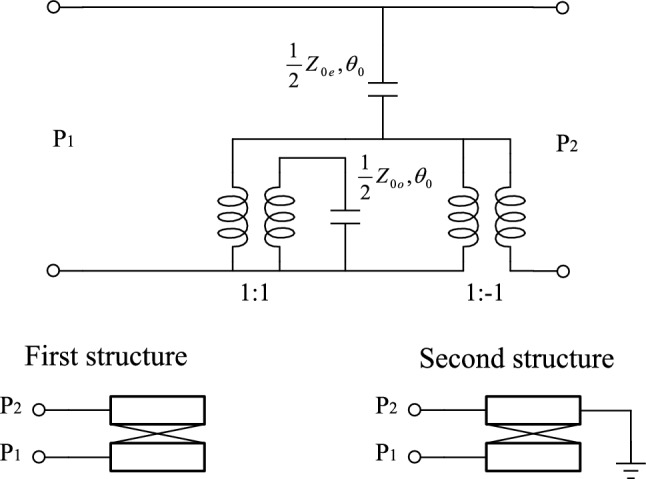
The high-impedance coupling model for first and second structures.

Figure 4a shows the equivalent model of the proposed DB-BPF based on the electrical length and impedance of the line. In general, three different lines are used in the structure of this filter from the point of view of impedance line: High-impedance lines with a thickness of 0.1 mm, low impedance lines with a thickness of 4 mm, and 50 Ω matching lines with a thickness of 1.2 mm. Based on the specified substrate for the amplifier, (substrate RO4003, ${{\varvec{\varepsilon}}}_{{\varvec{r}}}$=3.2, h = 20 mil, TanD = 0.0022) the thickness of the lines have been determined. As a result, the values of Z, Z1 and Z2 are known and equal to 50, 157 and 20 ohms respectively*.* High-impedance lines have a coupling effect and *ABCD* matrix was used to analyses them^19^. Since the proposed equivalent circuit has symmetry, even and odd mode analysis is chosen for it. The even mode of the equivalent circuit is depicted in Fig. 4b and the odd mode in Fig. 4c. The input impedance in odd mode ${{\varvec{Z}}}_{{\varvec{i}}{\varvec{n}}}^{{\varvec{o}}{\varvec{d}}{\varvec{d}}}$ is divided into three subsets ${{\varvec{Z}}}_{{\varvec{i}}{\varvec{n}}1}$, ${{\varvec{Z}}}_{{\varvec{i}}{\varvec{n}}2}$ and ${{\varvec{Z}}}_{{\varvec{i}}{\varvec{n}}3}$. Each subset is calculated as follows:

9 $${Z}_{{\text{in}}1}={Z}_{1}\frac{\left(-j{Z}_{2}{\text{cot}}{\theta }_{2}\right)+j{Z}_{1}{\text{tan}}{\theta }_{2}}{{Z}_{1}+j\left(-j{Z}_{2}{\text{cot}}{\theta }_{2}\right){\text{tan}}{\theta }_{2}}$$

10 $${Z}_{{\text{in}}2}=\frac{{A}_{{\text{T}}1}\left(j{Z}_{1}{\text{tan}}0.5{\theta }_{4}\right)+{B}_{{\text{T}}1}}{{C}_{{\text{T}}1}\left(j{Z}_{1}{\text{tan}}{0.5\theta }_{4}\right)+{D}_{{\text{T}}1}}$$

where ${A}_{{\text{T}}1}$,${B}_{{\text{T}}1}$,${C}_{{\text{T}}1}$ and ${D}_{{\text{T}}1}$, are parameters of *ABCD* matrix for coupled lines of T1. To calculate this matrix, first, the equivalent circuit of the coupled lines T1 has been introduced^21^, according to Fig. 5. Based on equivalent circuit, the *ABCD* matrix for T1 is calculated as follows:

11 $${T}_{1}=\left[\begin{array}{cc}{A}_{{\text{T}}1}& {B}_{{\text{T}}1}\\ {C}_{{\text{T}}1}& {D}_{{\text{T}}1}\end{array}\right]$$

12 $${A}_{{\text{T}}1}={D}_{{\text{T}}1}=\frac{{Z}_{0{\text{e}}1}{\text{cot}}{\theta }_{{\text{e}}1}+{Z}_{0{\text{o}}1}{\text{cot}}{\theta }_{{\text{o}}1}}{{Z}_{0{\text{e}}1}{\text{cot}}{\theta }_{{\text{e}}1}-{Z}_{0{\text{o}}1}{\text{cot}}{\theta }_{{\text{o}}1}}$$

13 $${B}_{{\text{T}}1}=-j\frac{{2Z}_{0{\text{e}}1}{Z}_{0{\text{o}}1}{\text{cot}}{\theta }_{{\text{e}}1}{\text{cot}}{\theta }_{{\text{o}}1}}{{Z}_{0{\text{e}}1}{\text{cot}}{\theta }_{{\text{e}}1}-{Z}_{0{\text{o}}1}{\text{cot}}{\theta }_{{\text{o}}1}}$$

14 $${C}_{{\text{T}}1}=j\frac{2}{{Z}_{0{\text{e}}1}{\text{cot}}{\theta }_{{\text{e}}1}-{Z}_{0{\text{o}}1}{\text{cot}}{\theta }_{{\text{o}}1}}$$

In the following, the odd mode calculation of input impedance:

15 $${Z}_{{\text{in}}3}=\frac{{A}_{{\text{Tx}}}\left(-j{Z}_{1}{\text{tan}}2{\theta }_{3}\right)+{B}_{{\text{Tx}}}}{{C}_{{\text{Tx}}}\left(-j{Z}_{1}{\text{tan}}2{\theta }_{3}\right)+{D}_{{\text{Tx}}}}$$

where ${A}_{{\text{Tx}}}$,${B}_{{\text{Tx}}}$,${C}_{{\text{Tx}}}$ and ${D}_{{\text{Tx}}}$ are parameters of *ABCD* matrix for ${T}_{{\text{X}}}$, which can be derived as:

16 $${T}_{{\text{X}}}={T}_{{\text{T}}2}\times {T}_{{\text{T}}3}=\left[\begin{array}{cc}{A}_{{\text{T}}2}& {B}_{{\text{T}}2}\\ {C}_{{\text{T}}2}& {D}_{{\text{T}}2}\end{array}\right]\times \left[\begin{array}{cc}{A}_{{\text{T}}3}& {B}_{{\text{T}}3}\\ {C}_{{\text{T}}3}& {D}_{{\text{T}}3}\end{array}\right]$$

17 $${T}_{{\text{T}}2}=\left\{\begin{array}{c}{A}_{{\text{T}}2}={D}_{{\text{T}}2}=\frac{{Z}_{0{\text{e}}2}{\text{cot}}{\theta }_{{\text{e}}2}+{Z}_{0{\text{o}}2}{\text{cot}}{\theta }_{{\text{o}}2}}{{Z}_{0{\text{e}}2}{\text{cot}}{\theta }_{{\text{e}}2}-{Z}_{0{\text{o}}2}{\text{cot}}{\theta }_{{\text{o}}2}}\\ {B}_{{\text{T}}2}=-j\frac{{2Z}_{0{\text{e}}2}{Z}_{0{\text{o}}2}{\text{cot}}{\theta }_{{\text{e}}2}{\text{cot}}{\theta }_{{\text{o}}2}}{{Z}_{0{\text{e}}2}{\text{cot}}{\theta }_{{\text{e}}2}-{Z}_{0{\text{o}}2}{\text{cot}}{\theta }_{{\text{o}}2}}\\ {C}_{{\text{T}}2}=j\frac{2}{{Z}_{0{\text{e}}2}{\text{cot}}{\theta }_{{\text{e}}2}-{Z}_{0{\text{o}}2}{\text{cot}}{\theta }_{{\text{o}}2}}\end{array}\right.$$

18 $${T}_{{\text{T}}3}=\left\{\begin{array}{c}{A}_{{\text{T}}2}=\frac{{Z}_{0e3}^{2}+{Z}_{0o3}^{2}-2{Z}_{0{\text{e}}3}{Z}_{0{\text{o}}3}{\text{csc}}{\theta }_{{\text{e}}3}{\text{csc}}{\theta }_{{\text{o}}3}({\text{cos}}{\theta }_{{\text{e}}3}{\text{cos}}{\theta }_{{\text{o}}3}+1)}{{Z}_{0e3}^{2}-{Z}_{0o3}^{2}}\\ {B}_{{\text{T}}2}=-j\frac{{2Z}_{0{\text{e}}3}{Z}_{0{\text{o}}3}({Z}_{0{\text{o}}3}{\text{cot}}{\theta }_{{\text{e}}3}+{Z}_{0{\text{e}}3}{\text{cot}}{\theta }_{{\text{o}}3})}{{Z}_{0e3}^{2}-{Z}_{0o3}^{2}}\\ \begin{array}{c}{C}_{{\text{T}}2}=-j\frac{2({Z}_{0{\text{e}}3}{\text{cot}}{\theta }_{{\text{e}}3}+{Z}_{0{\text{o}}3}{\text{cot}}{\theta }_{{\text{o}}3})}{{Z}_{0e3}^{2}-{Z}_{0o3}^{2}}\\ {D}_{{\text{T}}2}=\frac{{Z}_{0e3}^{2}+{Z}_{0o3}^{2}-2{Z}_{0{\text{e}}3}{Z}_{0{\text{o}}3}{\text{csc}}{\theta }_{{\text{e}}3}{\text{csc}}{\theta }_{{\text{o}}3}({\text{cos}}{\theta }_{{\text{e}}3}{\text{cos}}{\theta }_{{\text{o}}3}-1)}{{Z}_{0e3}^{2}-{Z}_{0o3}^{2}}\end{array}\end{array}\right.$$

As it is known, parameters of ${T}_{{\text{T}}3}$ are different from ${T}_{{\text{T}}2}$ because in odd mode, ${T}_{{\text{T}}3}$ structure is connected from one port to ground (Fig. 5, second structure). Next, ${Z}_{{\text{in}}}^{{\text{odd}}}$ is calculated as follows:

19 $${Z}_{{\text{in}}}^{{\text{odd}}}={Z}_{{\text{o}}}\frac{\left({Z}_{{\text{in}}1}||{Z}_{{\text{in}}2}||{Z}_{{\text{in}}3}\right)+j{Z}_{{\text{o}}}{\text{tan}}{\theta }_{{\text{o}}}}{{Z}_{{\text{o}}}+j\left({Z}_{{\text{in}}1}||{Z}_{{\text{in}}2}||{Z}_{{\text{in}}3}\right){\text{tan}}{\theta }_{{\text{o}}}}$$

As it is clear from (19), after calculating the input impedance of all three subsets of the odd mode, all three subsets are paralleled together, and then the total impedance is connected in series with the matching line. In the same way, for even mode, we have as follows:

The input impedance in the even mode is also divided into three subsets ${Z}_{{\text{in}}1}$, ${Z}_{{\text{in}}2}$ and ${Z}_{{\text{in}}3}$. Each subset is calculated as follows:

20 $${Z}_{{\text{in}}4}={Z}_{1}\frac{\left(-j{Z}_{2}{\text{cot}}{\theta }_{2}\right)+j{Z}_{1}{\text{tan}}{\theta }_{2}}{{Z}_{1}+j\left(-j{Z}_{2}{\text{cot}}{\theta }_{2}\right){\text{tan}}{\theta }_{2}}$$

21 $${Z}_{{\text{in}}5}=\frac{{A}_{{\text{T}}1}\left(-j{Z}_{1}{\text{cot}}0.5{\theta }_{4}\right)+{B}_{{\text{T}}1}}{{C}_{{\text{T}}1}\left(-j{Z}_{1}{\text{cot}}{0.5\theta }_{4}\right)+{D}_{{\text{T}}1}}$$

where ${A}_{{\text{T}}1}$,${B}_{{\text{T}}1}$,${C}_{{\text{T}}1}$ and ${D}_{{\text{T}}1}$, are parameters of *ABCD* matrix for coupled lines of T1, which has been reviewed before (according to Fig. 5, the first structure). Based on this equivalent circuit, the *ABCD* matrix for T1 is calculated as follows:

22 $${T}_{1}=\left[\begin{array}{cc}{A}_{{\text{T}}1}& {B}_{{\text{T}}1}\\ {C}_{{\text{T}}1}& {D}_{{\text{T}}1}\end{array}\right]$$

23 $${A}_{{\text{T}}1}={D}_{{\text{T}}1}=\frac{{Z}_{0{\text{e}}1}{\text{cot}}{\theta }_{{\text{e}}1}+{Z}_{0{\text{o}}1}{\text{cot}}{\theta }_{{\text{o}}1}}{{Z}_{0{\text{e}}1}{\text{cot}}{\theta }_{{\text{e}}1}-{Z}_{0{\text{o}}1}{\text{cot}}{\theta }_{{\text{o}}1}}$$

24 $${B}_{{\text{T}}1}=-j\frac{{2Z}_{0{\text{e}}1}{Z}_{0{\text{o}}1}{\text{cot}}{\theta }_{{\text{e}}1}{\text{cot}}{\theta }_{{\text{o}}1}}{{Z}_{0{\text{e}}1}{\text{cot}}{\theta }_{{\text{e}}1}-{Z}_{0{\text{o}}1}{\text{cot}}{\theta }_{{\text{o}}1}}$$

25 $${C}_{{\text{T}}1}=j\frac{2}{{Z}_{0{\text{e}}1}{\text{cot}}{\theta }_{{\text{e}}1}-{Z}_{0{\text{o}}1}{\text{cot}}{\theta }_{{\text{o}}1}}$$

In the following, we have the calculation of input impedance in even mode:

26 $${Z}_{{\text{in}}6}=\frac{{A}_{{\text{Tx}}}\left(-j{Z}_{1}{\text{tan}}2{\theta }_{3}\right)+{B}_{{\text{Tx}}}}{{C}_{{\text{Tx}}}\left(-j{Z}_{1}{\text{tan}}2{\theta }_{3}\right)+{D}_{{\text{Tx}}}}$$

where ${A}_{{\text{Tx}}}$,${B}_{{\text{Tx}}}$,${C}_{{\text{Tx}}}$ and ${D}_{{\text{Tx}}}$ are parameters of *ABCD* matrix for ${T}_{{\text{X}}}$, which can be derived as:

27 $${T}_{{\text{X}}}={T}_{{\text{T}}2}\times {T}_{{\text{T}}3}=\left[\begin{array}{cc}{A}_{{\text{T}}2}& {B}_{{\text{T}}2}\\ {C}_{{\text{T}}2}& {D}_{{\text{T}}2}\end{array}\right]\times \left[\begin{array}{cc}{A}_{{\text{T}}3}& {B}_{{\text{T}}3}\\ {C}_{{\text{T}}3}& {D}_{{\text{T}}3}\end{array}\right]$$

28 $${T}_{{\text{T}}2}=\left\{\begin{array}{c}{A}_{{\text{T}}2}={D}_{{\text{T}}2}=\frac{{Z}_{0{\text{e}}2}{\text{cot}}{\theta }_{{\text{e}}2}+{Z}_{0{\text{o}}2}{\text{cot}}{\theta }_{{\text{o}}2}}{{Z}_{0{\text{e}}2}{\text{cot}}{\theta }_{{\text{e}}2}-{Z}_{0{\text{o}}2}{\text{cot}}{\theta }_{{\text{o}}2}}\\ {B}_{{\text{T}}2}=-j\frac{{2Z}_{0{\text{e}}2}{Z}_{0{\text{o}}2}{\text{cot}}{\theta }_{{\text{e}}2}{\text{cot}}{\theta }_{{\text{o}}2}}{{Z}_{0{\text{e}}2}{\text{cot}}{\theta }_{{\text{e}}2}-{Z}_{0{\text{o}}2}{\text{cot}}{\theta }_{{\text{o}}2}}\\ {C}_{{\text{T}}2}=j\frac{2}{{Z}_{0{\text{e}}2}{\text{cot}}{\theta }_{{\text{e}}2}-{Z}_{0{\text{o}}2}{\text{cot}}{\theta }_{{\text{o}}2}}\end{array}\right.$$

29 $${T}_{{\text{T}}3}=\left\{\begin{array}{c}{A}_{{\text{T}}3}={D}_{{\text{T}}3}=\frac{{Z}_{0{\text{e}}3}{\text{cot}}{\theta }_{{\text{e}}3}+{Z}_{0{\text{o}}3}{\text{cot}}{\theta }_{{\text{o}}3}}{{Z}_{0{\text{e}}3}{\text{cot}}{\theta }_{{\text{e}}3}-{Z}_{0{\text{o}}3}{\text{cot}}{\theta }_{{\text{o}}3}}\\ {B}_{{\text{T}}3}=-j\frac{{2Z}_{0{\text{e}}3}{Z}_{0{\text{o}}3}{\text{cot}}{\theta }_{{\text{e}}3}{\text{cot}}{\theta }_{{\text{o}}3}}{{Z}_{0{\text{e}}3}{\text{cot}}{\theta }_{{\text{e}}3}-{Z}_{0{\text{o}}3}{\text{cot}}{\theta }_{{\text{o}}3}}\\ {C}_{{\text{T}}3}=j\frac{2}{{Z}_{0{\text{e}}3}{\text{cot}}{\theta }_{{\text{e}}3}-{Z}_{0{\text{o}}3}{\text{cot}}{\theta }_{{\text{o}}3}}\end{array}\right.$$

As it is known, parameters of ${T}_{{\text{T}}3}$ are different than odd mode, because in even mode, ${T}_{{\text{T}}3}$ structure does not have ground port. Next, ${Z}_{{\text{in}}}^{{\text{even}}}$ is calculated as follows:

30 $${Z}_{{\text{in}}}^{{\text{even}}}={Z}_{{\text{o}}}\frac{\left({Z}_{{\text{in}}4}||{Z}_{{\text{in}}5}||{Z}_{{\text{in}}6}\right)+j{Z}_{{\text{o}}}{\text{tan}}{\theta }_{{\text{o}}}}{{Z}_{{\text{o}}}+j\left({Z}_{{\text{in}}4}||{Z}_{{\text{in}}5}||{Z}_{{\text{in}}6}\right){\text{tan}}{\theta }_{{\text{o}}}}$$

As it is clear from (30), after calculating the input impedance of all three subsets of the odd mode, all three subsets are paralleled together, and then the total impedance is connected in series with the matching line.

After calculating the input impedances in odd and even modes, the important s-parameters can be obtained from the following equations:

31 $${{\text{S}}}_{21}=\frac{\left({Z}_{{\text{in}}}^{{\text{even}}}-{Z}_{{\text{in}}}^{{\text{odd}}}\right){Z}_{0}}{\left({Z}_{{\text{in}}}^{{\text{even}}}+{Z}_{0}\right)\cdot \left({Z}_{{\text{in}}}^{{\text{odd}}}+{Z}_{0}\right)}$$

32 $${{\text{S}}}_{11}=\frac{{Z}_{{\text{in}}}^{{\text{even}}}{Z}_{{\text{in}}}^{{\text{odd}}}- {{Z}_{0}}^{2}}{\left({Z}_{{\text{in}}}^{{\text{even}}}+{Z}_{0}\right)\cdot \left({Z}_{{\text{in}}}^{{\text{odd}}}+{Z}_{0}\right)}$$

As mentioned earlier, high-impedance lines, low-impedance lines and matching lines are used in the proposed filter structure. As a result, the values of Z = 50, Z_1_ = 132 and Z_2_ = 20 Ω have been determined. It was also mentioned that the coupling lines are composed of high-impedance lines symmetrically, as a result ${Z}_{0{\text{oi}}}={Z}_{0{\text{ei}}}({\text{i}}=\mathrm{1,2},3)= 132$ ohm. Since the beginning and end lines of the circuit are equivalent to the characteristic Z, ${\theta }_{1}$ of the matching lines, the value of ${\theta }_{1}$ does not affect the changes in the frequency location of transmission zeros and poles of the filter. Finally, there are three unknowns in the equations: ${\theta }_{2}$, ${\theta }_{3}$ and ${\theta }_{4}$. These three variables are obtained by using conditions of transmission zero and poles. Since the DB-BPF has two passbands, it is necessary to create a transmission poles for each passband, in other words, ${{\text{S}}}_{21}\left(@2.1{\text{GHZz}}\right)=1(0 {\text{dB}})$ and ${{\text{S}}}_{21}\left(@2.9{\text{GHZz}}\right)=1(0 {\text{dB}})$. Next, in order to have sharp roll-off, two transmission zeros are needed at the end of the lower stopband and the beginning of the upper stopband. These two transmission zeros are considered at 1.5 and 3.5 GHz. So two more conditions are created: ${{\text{S}}}_{21}\left(@1.5{\text{GHZz}}\right)=0(-\infty {\text{dB}})$ and ${{\text{S}}}_{21}\left(@2.9{\text{GHZz}}\right)=0(-\infty {\text{dB}})$. To create proper isolation between the two bands, a transmission zero between the two bands is included at the frequency of 2.5 GHz, so its condition is also equal to: ${{\text{S}}}_{21}\left(@2.5{\text{GHZz}}\right)=0(-\infty {\text{dB}})$. By applying the mentioned conditions, values of ${\theta }_{2}$, ${\theta }_{3}$ and ${\theta }_{4}$ are obtained. From S_11_ and S_21_ equations, and resonant conditions of transmission zeros and poles, the electrical length of the lines is calculated. The calculated values in the ADS simulator have been optimized to achieve the best results, by using gradient optimization type. Microstrip elements are then designed in the layout section of ADS, based on the optimized values. At this stage, the high-impedance lines are meandered as much as possible to minimize the overall size of the filter. In this regard, Table 1 shows the calculated and optimized values in ADS software.

**Table 1:** Calculated and optimized values of equivalent model of the proposed DB-BPF.

Parameters	${{\varvec{\theta}}}_{\mathbf{o}}$	${{\varvec{\theta}}}_{1}$	${{\varvec{\theta}}}_{\mathbf{o}1}$	${{\varvec{\theta}}}_{2}$	${{\varvec{\theta}}}_{\mathbf{o}2}$	${{\varvec{\theta}}}_{3}$	${{\varvec{\theta}}}_{\mathbf{o}3}$	${{\varvec{\theta}}}_{\mathbf{o}4}$
Calculated value	14.02°	43.75°	53.33°	25.21°	20.11°	12.94 ^o^	26.15^o^	26.88°
Optimized value	14.25°	45.01°	52.52°	22.11°	19.51°	12.75°	25.89°	26.26°

### Design process of DB-BPF

The overall design process of the DB-BPF involves a detailed examination of its various components, with the role of each part in the overall structure determined based on the final response of the filter.

**Figure 6:**
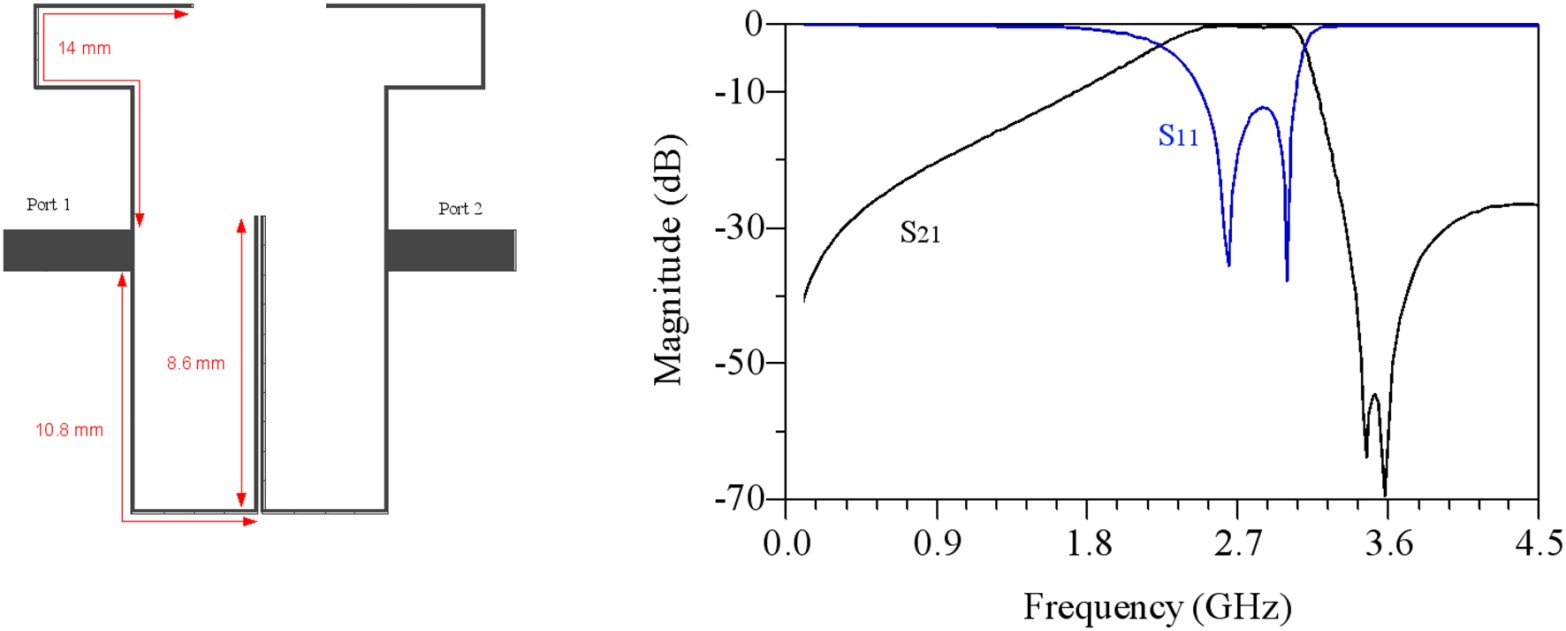
Structure and simulated results of single-band bandpass filter with high impedance lines.

**Figure 7:**
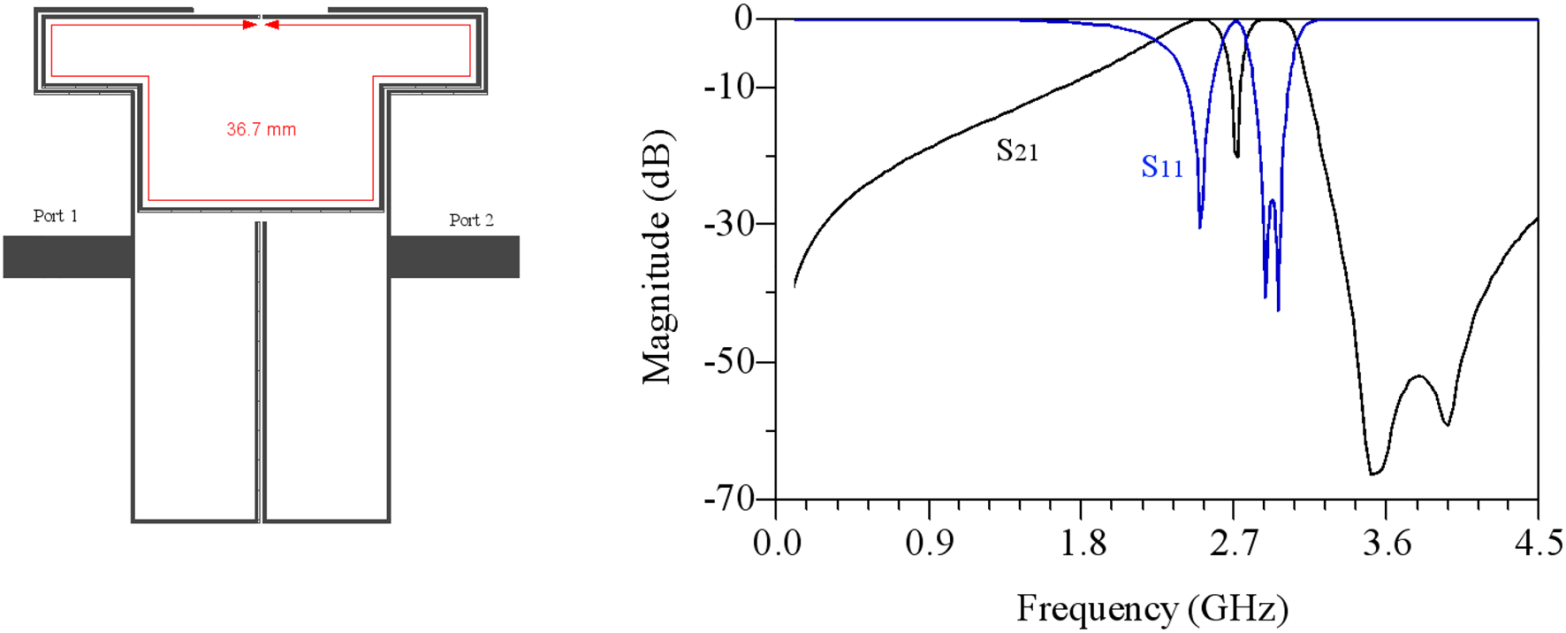
Dual-band bandpass filter with the addition of high-impedance lines.

**Figure 8:**
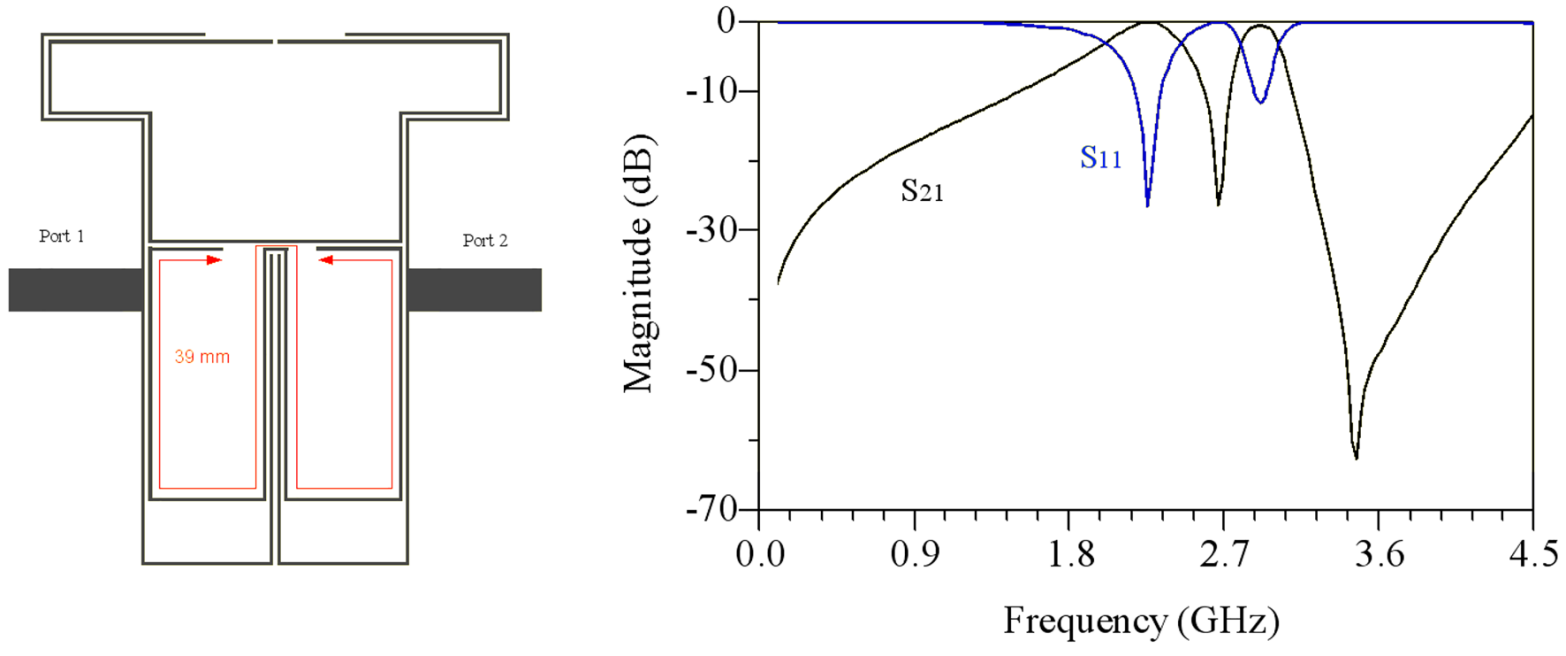
structure and simulation results of BPF with adjustment of the first passband and increased isolation.

**Figure 9:**
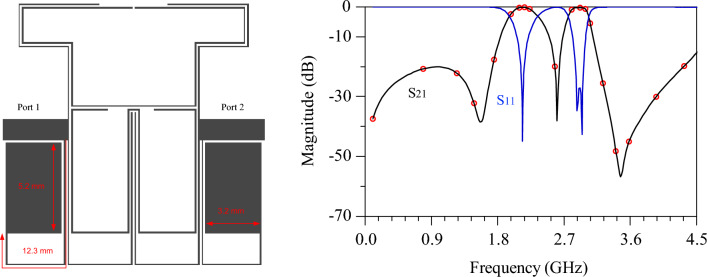
Proposed DB-BPF with its simulated responses.

- Single-band BPF: Initially, the structure comprises open-ended high-impedance lines and double-coupled lines, as illustrated in Fig. 6. This configuration creates a passband from 2.5 to 3 GHz with an insertion loss of less than 1 dB. However, the roll-off in this band is found to be inadequate.
- Dual-band BPF: To address the limitations of the single-band filter, high-impedance lines are added to the open-end lines, and a coupling effect is introduced in the open-end lines. This modification results in the creation of a transmission zero in the middle of the passband of the previous filter, as depicted in Fig. 7. The dual-band filter derived from this process exhibits passbands at the central frequencies of 2.5 and 2.9 GHz, providing an improved response.
- Increasing the isolation between the two bands: To reduce the frequency location of the first passband of the filter, high-impedance lines have been incorporated to enhance the coupling effect, as illustrated in Fig. 8. Simulation responses in Fig. 8 indicate that the central frequency of the first passband is 2.25 GHz, and the second band is at 2.92 GHz. However, the challenge of achieving a sharp response persists in the first band.
- Improving the roll-off: Addressing the need for an enhanced roll-off and an extended lower stopband, a transmission zero is introduced using an elliptical resonator. This resonator comprises an open-ended low-impedance line connected to a high-impedance line. The final structure of the filter is presented in Fig. 9. According to Fig. 9, the central frequency of the first band is 2.1 GHz with a bandwidth ranging from 1.97 to 2.34 GHz. Similarly, the central frequency of the second band is 2.9 GHz, and its bandwidth spans from 2.76 to 3.03 GHz. In essence, both bands exhibit a bandwidth of 0.37 GHz each. The insertion and return losses at the central frequencies of the first and second bands are recorded at 0.13 dB, 45 dB, 0.28 dB, and 28.2 dB, respectively.

The design procedure of HCC (WB-BC and ECR) and DB-BPF are specified as follows:

- WB-BC Design Procedure: In the design of this circuit, three microstrip lines are employed, with their lengths adjusted to resonate at three distinct frequencies. The electrical lengths are specifically set for each line to achieve resonance at the following frequencies: The TL1 line alone is tuned for resonance at the second frequency of the power amplifier, 2.91 GHz. The TL1 line in conjunction with TL2 is configured for resonance at the first frequency of the power amplifier, 2.1 GHz. Finally, the TL1 line combined with TL3 is tuned to resonate at a lower frequency of 1.5 GHz.
- ECR Design Procedure: Initially, the input impedance is calculated by utilizing steps (1) through (8), relying on the ABCD matrix of the complete structure. Subsequently, under resonance conditions for the second harmonics at frequencies of 4.2 and 5.82 GHz, the parameters of the proposed structure are determined. The values derived through ADS software are optimized and subsequently applied in the layout section employing microstrip elements.
- DB-BPF Design Procedure: Initially, the input impedance in both even and odd modes is determined for the proposed equivalent model, employing steps (9) through (32). Subsequently, the parameters of the equivalent model are calculated based on resonance conditions for the transmission poles and zeros. Following this, the calculated values are optimized using ADS software and applied in the layout section through microstrip elements. During this stage, high impedance lines are strategically employed in the DBBPF to reduce the overall size of the filter.

## Dual-band PA design

To validate the proposed approach, a dual-band PA is fabricated utilizing the LDMOS AFT27S006N transistor and the proposed OMN on an RO4003 substrate with characteristics of ${\varepsilon }_{r}$=3.38, tanD = 0.0022, and thickness = 20 mil. The transistor is biased with a voltage setting of V_DD_ = 28 V and V_GG_ = 1.7 V. The DC blocking capacitor used in the design has a capacitance of 100 pF, while the bypass and DC coupling capacitors have capacitances of 30 pF and 1 uF, respectively.

**Figure 10:**
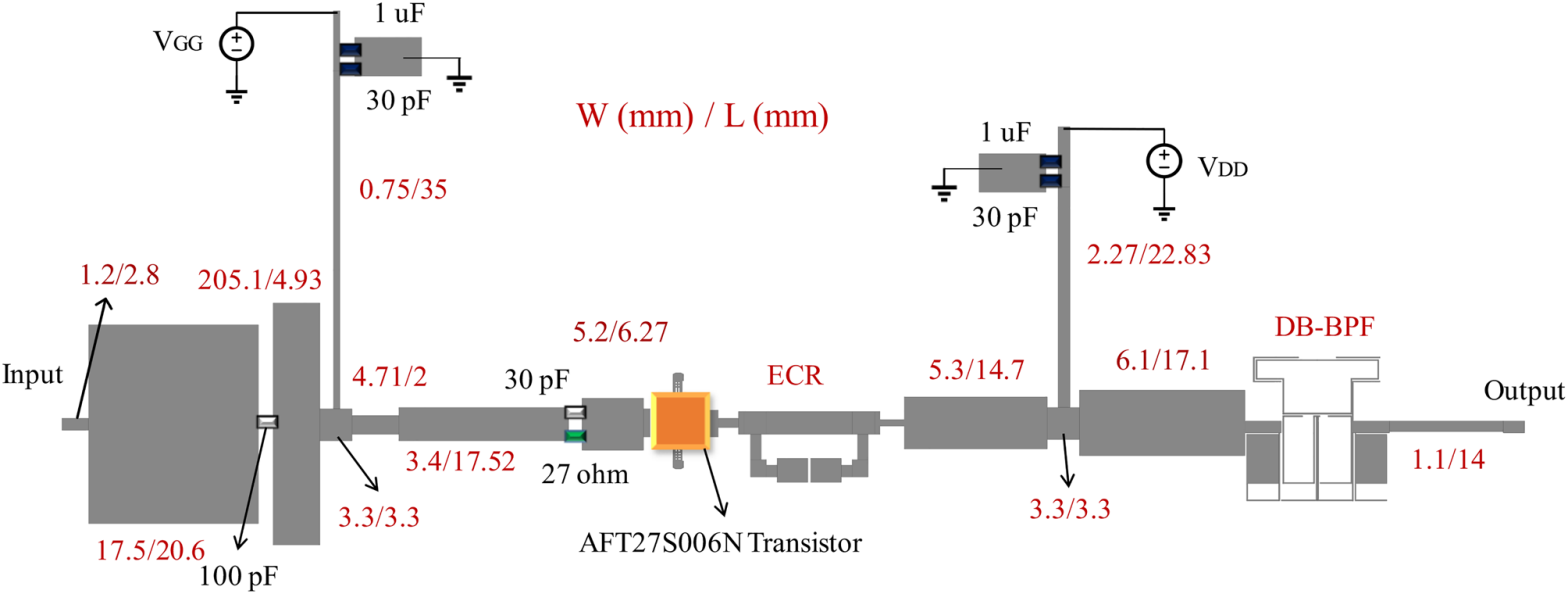
Schematics of the proposed DB-PA.

**Figure 11:**
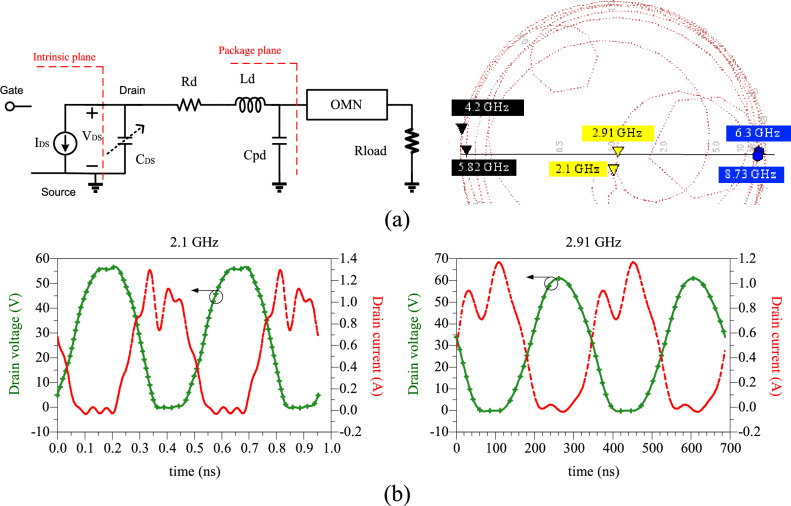
Simulation results of the proposed dual-band amplifier, (**a**) the input impedance of OMN on the Smith chart, and (**b**) drain voltage/current waveforms at i-gen plane of transistor.

The stability circuit is composed of a 27-Ω resistor in parallel with a 30 pF capacitor. All components employed are Surface Mount Devices (SMD) and adhere to Murata models, ensuring suitability for operation at microwave frequencies. The structure of the proposed PA is illustrated in Fig. 10. Simulation results for this amplifier, encompassing OMN input impedance and drain current/voltage waveforms from the I-gen plane of the transistor's perspective, are presented in Fig. 11.

The lumped elements used in the proposed PA include the following: The 27-Ω resistor is parallel to the 30 pF capacitor in the circuit, serving to enhance the stability of the PA. Additionally, a 100 pF Murata high-frequency capacitor is utilized as a DC block capacitor to prevent the leakage of DC signals into the AC source. Moreover, high-frequency 22 pF Murata capacitors function as bypass capacitors. Lastly, high-frequency 1 uF Murata capacitors, acting as decoupling capacitors, safeguard the circuit against abrupt changes in the DC signal.

### The simulation and measurement results

**Figure 12:**
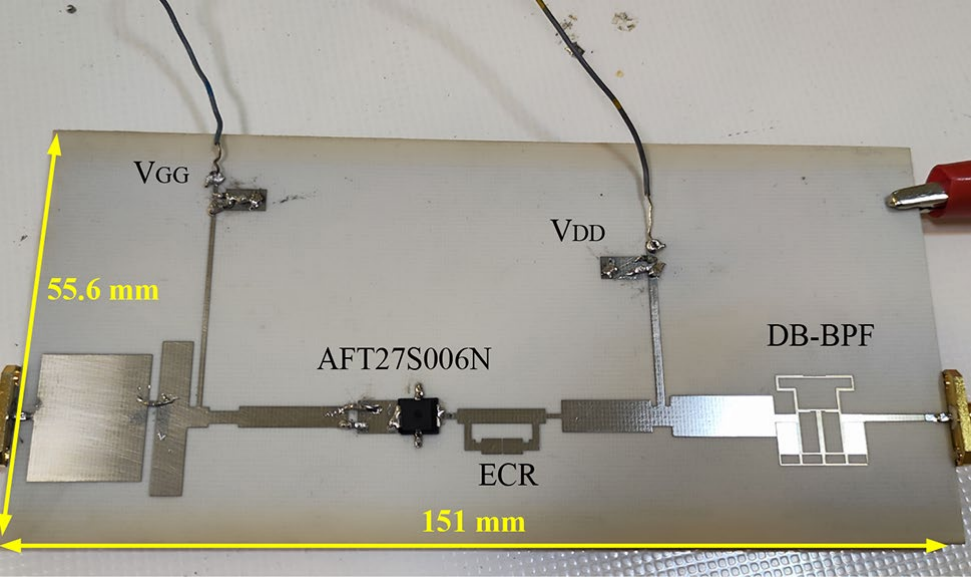
Photograph of the fabricated DB PA.

**Figure 13:**
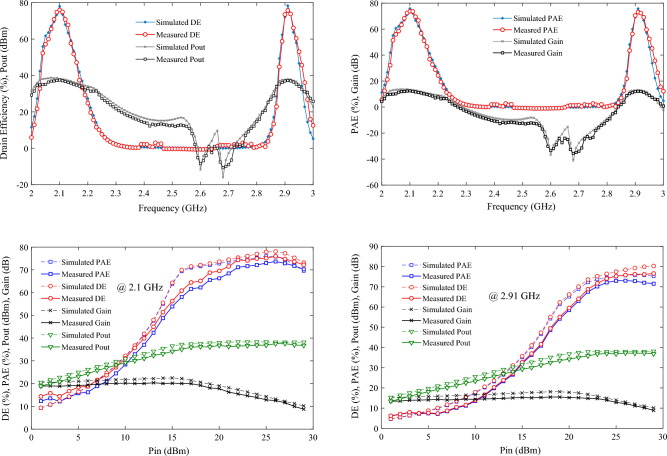
The large-signal results of all parameters in terms of frequency as well as input power.

**Figure 14:**
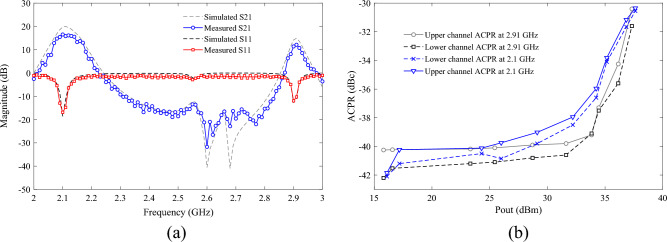
(**a**) The measured and simulated small-signal and (**b**) ACPR for output power at 2.1 and 2.91 GHz of proposed PA.

The large-signal performance of the proposed PA is evaluated using the R&S SMA100B RF and Microwave Analog Signal Generator, along with the R&S HMS3010 Spectrum Analyzer. In Fig. 12, a photograph of the fabricated PA is presented, while Fig. 12 showcases the large-signal results from both simulation and measurement, demonstrating good agreement. According to Fig. 13, the maximum measured efficiency at 3 dB gain compression is 75.98% and 75.73% at the operating frequencies of 2.1 and 2.91 GHz, respectively. Consequently, the measured Power-Added Efficiency (PAE) values are 73.68% and 73.03% at the operating frequencies of 2.1 and 2.91 GHz, respectively. The Output Power (Pout) values within the maximum range are 37.5 and 37.24 dBm at frequencies of 2.1 and 2.91 GHz, respectively, for an input power of 25 dBm. This indicates a power gain of 12.5 dB at 2.1 GHz and 12.24 dB at 2.91 GHz. Figure 14a depicts the small-signal results of the proposed amplifier, obtained using the Network Analyzer N9917A. These results indicate a voltage gain (S_21_) of 16.5 dB at 2.1 GHz and 12.13 dB at 2.91 GHz. At these frequencies, the input matching or S_11_ values are − 16.8 and − 12 dB, respectively. The input matching and voltage gain at the operational frequencies of the amplifier demonstrate its appropriate performance.

To test the linearity of the proposed dual-band PA, the PA is subjected to a Wideband Code Division Multiple Access (WCDMA) signal with a 5 MHz offset frequency and a peak-to-average power ratio (PAPR) of approximately 6.5 dB at frequencies of 2.1 and 2.91 GHz. The adjacent channel power ratio (ACPR) in the lower and upper channels is illustrated in Fig. 14b. As depicted in this figure, for the average output power ranging from 16.1 to 37.22 dBm at 2.1 GHz and for Pout ranging from 15.81 to 37.32 dBm at 2.91 GHz, ACPR in both channels varies between − 42.1 to − 30.35 dBc and − 42.2 to − 30.4, respectively. The ACPR values at both frequencies are observed to be lower than − 30 dBc, indicating excellent linearity performance.

### Comparison and discussion

As evident, the simulated and measured results are very close to each other in the two operating bands of the amplifier, which demonstrates the excellent performance of the proposed amplifier. Additionally, the measured responses show that the DB-BPF used in PA effectively isolates and controls efficiency and power gain within the two operating bands, even though the operating bands are close to each other. The narrow bandwidth of DB-BPF passbands do not allow the passage of interfering signals and unwanted harmonics, and the DC blocking capacitor between the amplifier and the load has been eliminated. As shown in Fig. 11, the overall size of the amplifier, are 151 × 55.6 mm^2^, which are very suitable compared to previous works. Table 2 compares the output results of the proposed amplifier with previous works. According to Table 2, the proposed amplifier outperforms previous works in terms of efficiency, dimensions, and output power.

**Table 2:** Comparison of measured results of the proposed DB-PA with previous works (*a*: PAE value).

Reference	Transistor	VDD (V)	*f*_1_/*f*_2_ (GHz)	DE (%)	Pout (dBm)	Gain (dB)	Design method
^1^	GaN HEMT	28	1.62/2.08	71.5/73 ^*a*^	40.2/40	–	DB harmonic-tuned
^2^	LDMOS	25	0.7/1.9	76.3/74.9	39.85/38.22	14.85/13.22	BSF, bias and compensator circuits
^3^	GaN HEMT	28	1.72/2.14	74.9/75.5 ^*a*^	40.5/40.9	–	DB-HCN and compensation circuit
^7^	GaN HEMT	28	0.7/1.75	72.5/70.5 ^*a*^	40.5/41	16/13	Dynamical continuous-mode criterions
^8^	GaN HEMT	28	2.6/3.5	76.7/72.8	42.4/41.1	11.5/10.5	Harmonic turning and RTF
^10^	GaN HEMT	28	2.45/5.76	62.9/61.7	39/35	11/7	Distributed element-based load network
^11^	GaN HEMT	28	1/2.3	72.474.1	41.6/42.1	11.6/11.2	DB coupler
^15^	GaN HEMT	28	2.45/3.3	53/46	33/32.5	10/9	Concurrent DB harmonic tuned
^16^	GaN HEMT	28	2.6/3.5	71/64	44.6/43.5	–	DB impedance transformer and reactance compensation network
^17^	0.1-um GaAS	7	6/16	55/53	26/25.5	14.9/9	Harmonic terminations
^18^	GaN HEMT	28	1.8/2.4	64/54	43/43	10/10	Concurrent DB Doherty
This work	LDMOS	28	2.1/2.91	75.98/75.73	37.5/37.24	12.5/12.24	DB-BPF, HCC

According to the measured results, the advantages of the proposed design compared to the previous works are as follows: high efficiency, closeness of the proposed amplifier performance in two bands, reduction of the overal size, proper isolation between two operational bands, considering that the operating bands are very close to each other. As a result, the proposed PA can be used for S-band applications, such as the LTE 2100 mobile radio standard (2.1 GHz with 70 MHz bandwidth) and WLAN (2.91 GHz with 80 MHz bandwidth).

### Review of novelties

The novelties of the proposed design include a new method for designing dual-band power amplifiers (PAs), dual-band bandpass filters (DB-BPFs), and dual-band harmonic control circuits (HCCs). These innovations are particularly tailored for dual-band PAs when the operating bands are in close proximity, necessitating effective isolation between the two bands.

Initially, a novel structure for dual-band power amplifier design using lumped components is introduced. Subsequently, various components of this circuit are replaced with microstrip elements and circuits suitable for microwave applications. Given the nature of the dual-band PA, operating with closely located frequency bands requiring isolation, a new DB-BPF has been developed. The DB-BPF exhibits well-defined lower and upper stopbands for suppressing unwanted harmonics. The creation of a transmission zero between the two bands enhances isolation with a high level of suppression. The DB-BPF features transmission poles at the operational frequencies of the amplifier, minimizing insertion and return losses at these frequencies to reduce losses and improve efficiency.

The introduced dual-band HCC circuit relies on two wideband bias circuits (WB-BC) and elliptically coupled resonators (ECR). As the operating frequencies are close, a wideband bias circuit based on three microstrip lines with a T-shaped structure is introduced for the first time to prevent AC signal leakage into the DC source. The ECR, leveraging the coupling effect between low-impedance lines, generates two transmission zeros in the second harmonics for PAs, effectively controlling the second harmonics. Ultimately, the proposed circuits, each featuring a new design and structure, contribute to increased efficiency and output power in dual-band PAs with closely located operational bands. The achieved excellent isolation sets apart these dual-band PAs from wideband PAs, making them suitable for dual-band applications.

## Conclusion

This research introduces a novel design approach for dual-band PAs based on a DB-BPF. The circuit architecture of this proposed method is structured using four resonators and RFC inductors. The first two resonators are designed to regulate the second harmonics, while the third and fourth resonators serve as a harmonic blocker, allowing only the primary signals to pass through to the load. The resulting dual-band PA, referred to as the proposed OMN, is formed by replacing all components with circuits constructed using microstrip elements. The proposed OMN integrates an improved DB-BPF, ECR, and a DB HCC. Additionally, a compensator line has been introduced to match the transistor with the suggested OMN. Subsequently, a dual-band PA has been fabricated and measured at two operational frequencies of 2.1 and 2.91 GHz. The comparison between measurement and simulation results demonstrates a close match, validating the precision of the proposed design. Leveraging these advantages, the suggested design approach has the potential to enhance the efficiency of PAs utilized in Multistandard transceiver systems.

## Data Availability

The calculated results during the current study are available from the corresponding author on reasonable request.
